# Acute Effects of Three Cooked Non-Cereal Starchy Foods on Postprandial Glycemic Responses and in Vitro Carbohydrate Digestion in Comparison with Whole Grains: A Randomized Trial

**DOI:** 10.3390/nu11030634

**Published:** 2019-03-15

**Authors:** Ruixin Zhu, Zhihong Fan, Yue Han, Shuang Li, Guojing Li, Linlin Wang, Ting Ye, Wenqi Zhao

**Affiliations:** 1 Beijing Advanced Innovation Centre for Food Nutrition and Human Health, College of Food Science & Nutritional Engineering, China Agricultural University, Beijing 100083, China; zhuruixin07@126.com (R.Z.); lsshuangshuang@163.com (S.L.); yingfengdelei@126.com (G.L.); lynn9523@126.com (L.W.); tinacau@126.com (T.Y.); zhaowenqi@cau.edu.cn (W.Z.); 2 College of Biological Sciences, China Agricultural University, Beijing 100094, China; hanyue@cau.edu.cn

**Keywords:** lotus seed, adzuki bean, starchy food, glycemic response, in vitro carbohydrate digestion

## Abstract

Plant origin, processing, and domestic preparation may affect the postprandial glycemic response (PGR) of starchy foods. The objective of this study was to examine the possibility of integrating domestically cooked non-cereal starchy foods commonly consumed in Northeast Asia into glycemic management diet, and compare their glycemic characteristics with those of waxy and non-waxy whole grains and starchy beans. In a randomized crossover trial, ten healthy subjects consumed dried lily bulb (LB), lotus seed (LS), adlay (AD), waxy black rice (BR), millet (MI), and adzuki bean (AB), pre-soaked and each cooked for two time durations. Acute PGR tests and in vitro carbohydrate digestion were carried out for each test food. Both the LS and AB meals achieved low glycemic index (GI 21–51), while the other starchy foods failed to show significant difference with rice (GI 83–109). The hydrolysis indexes of LS and AB were 37.7%–61.1%, significantly lower than other test foods. The in vitro tests indicated that pre-soaking resulted in high rapidly digestible starch (RDS) and low resistant starch (RS). Careful choice of whole grain materials, minimized pre-soaking, and moderate cooking may be critical factors for successful postprandial glycemic management for diabetic and pre-diabetic.

## 1. Introduction

The consumption of refined rice, which is the staple food in most Asian countries, is associated with increased risk of type 2 diabetes (T2D) [1,2,3,4,5] and is prone to cause hyperglycemia after meals, even in healthy adults [6], whereas whole grains and pulses were reported to be inversely associated with risk of T2D [1,2,3,7,8,9,10,11] and relatively mild postprandial glycemic responses (GR) [7]. There is accumulated evidence that the substitution of high-glycemic index (GI) refined grain products with low-GI carbohydrate food such as whole grains [12] and legumes [13] could improve insulin sensitivity and resistance, and thus be beneficial to the prevention of many metabolic syndromes, such as obesity, hypertension, non-alcoholic fatty liver disease, polycystic ovary syndrome, hypertriglyceridemia, and cardiovascular disease [14]. The dietary guidelines of America [15], China [16], Singapore [17], and Japan [18] recommended the incorporation of whole grains and legumes into diet as a source of carbohydrate.

Since the chronic diseases associated with hyperglycemia represent leading public health concerns worldwide, it is relevant to identify carbohydrate food materials that may contribute to daily blood glucose management. In addition to cereals and pulses, there are several starchy food materials in traditional Asian diets, such as the lotus seed, adlay (Coix seed, Job’s-tears), and dried lily bulb. However, the postprandial GR of them is rarely reported [19].

On the other hand, being a whole grain is not a guarantee of being a low-GI food material. The decreased GR of whole grains and pulses could partly be attributed to the slow release of glucose during the digestion process, which may be explained by the content of resistant starch (RS) and slow-digested starch (SDS) [20]. However, there are reports that some varieties of waxy whole grains could elicit high GR comparable to glucose [21], and domestic preparation such as prolonged boiling, baking, frying, and roasting has substantial impact on the GR of reported low-GI starchy grains [22,23].

In this study, we tried to investigate the possibility of integrating the three non-cereal starchy food, i.e., the lotus seed, adlay, and dried lily bulb into a glycemic management diet, and compare their glycemic characteristics with those of non-waxy whole grains (millet), waxy whole grains (waxy black rice) and adzuki bean, which are commonly consumed in Northeast Asia, while taking the pre-soaking and cooking duration into account. We assumed that domestically cooked lotus seed, adlay, and dried lily bulb might have lower GR and RS compared with waxy black rice and polished rice, and can be used as low-GI ingredients as whole grains in Oriental staple food. The acute GR and the GI value as well as the in vitro carbohydrate digestion of the abovementioned samples were measured.

## 2. Materials and Methods

### 2.1. Materials

All seven materials were bought in the local supermarket. Waxy black rice (*Oryza sativa* Linn. spp.) and adlay (*Coix lacryma-jobi* Linn.) were produced in Daqing, Heilongjiang Province, China. Foxtail millet (*Setaria italica*), and adzuki bean (*Vigna angularis* var. angularis) were cultivated in Dalian, Liaoning Province, China. Dried lily bulb (*Lilium brownii* var. viridulum Baker) was from Lanzhou, Gansu Province, China. Lotus seed (*Nelumbo nucifera* Gaertn.) was produced in Honghu, Hubei Province, China. White rice (*Oryza sativa* spp. *japonica*) was cultivated and milled in Jilin, Jilin Province, China, which was used as reference food.

### 2.2. Preparation of Test Foods

The test foods were pre-prepared as follows. Each test food, which contains 50.0 g of available carbohydrate, including 75.0 g of adlay (AD), 72.3 g of millet (MI), 66.1 g of waxy black rice (BR), 77.6 g of lotus seed (LS), 74.0 g of dried lily bulb (LB), and 83.1 g of adzuki bean (AB), was soaked in small plastic containers and placed in the refrigerator at 4 °C for 12 h. The ratio of grains and water was 1.0:1.5.

On the test day, the containers containing soaked adzuki bean were placed in a steamer and cooked under normal pressure at 1000 W for 40 min and 70 min, respectively; the other test foods were steamed under normal pressure at of 1000 W for 30 min and 60 min, respectively. 66.1 g of white rice (WR) containing 50 g of available carbohydrate was steamed under normal pressure at of 1000 W for 30 min. All these foods were served to subjects at approximately 40 °C. Fifty grams of water-free glucose was dissolved in 200 mL of water at the room temperature. The glucose solution and cooked WR were prepared as dual reference foods. The composition of test foods and reference foods is shown in Table 1.

### 2.3. Blood Glucose Test

#### 2.3.1. Study Subjects

Only young women were recruited as subjects, because during the glycemic response test, a satiety test was conducted as well. It might cause extra error if male were included in the satiety test (see Supplementary Materials), as males and females usually differ considerately in appetite. Young female individuals aged 18~26 were recruited via online advertisements and posters based on following criteria: (1) normal blood glucose level; (2) body mass index (BMI):18~25 kg/m^2^; (3) not being on a diet or gaining weight deliberately in the past 3 months; (4) not breakfast-skippers and regularly consuming three meals; (5) not taking prescription medicines or nutritional supplements for 3 months prior to the study; (6) non-smoker; (7) no food allergy; (8) no metabolic disease and family history; (9) no gastrointestinal disease and (10) not in pregnancy or lactation period. The sample size was calculated with the PASS 11 Power Analysis and Sample Size (NCSS, Kaysville, UT, USA). It found that a blood glucose test with ten subjects would have 80% power to examine a difference (*p* < 0.05) of 20 mmol/L·2 h in iAUC. Ten potential volunteers who met aforementioned requirements were involved in an oral glucose tolerance test (OGTT). All the subjects passed the OGTT test and signed the consent forms. The study was conducted at the Beijing Advanced Innovation Centre for Food Nutrition and Human Health, College of Food Science and Nutritional Engineering, China Agricultural University, performed according to the Declaration of Helsinki (1964) and its later amendments, with the study protocol approved by the Human Study Ethics Committee of China Agricultural University (ethics number 2016012).

#### 2.3.2. Experimental Procedures

The study protocol was in accordance with internationally recognized GI methodology recommended by the Food and Agriculture Organization and the World Health Organization. This study was based on an undouble-blind, randomized crossover design and subjects ate all the test foods on 16 separate mornings, including 12 times for testing starchy foods, and twice for glucose and cooked rice references, respectively. Participants were recruited to this study by using a single allocate ratio. Sequentially numbered containers were used to implement the random allocation sequence. The wash-out period between each test session was no less than five days. During the test sessions, the subjects were asked to refrain from alcoholic beverages, strenuous exercise, and inadequate sleep. The trial days were arranged so to avoid the menstrual period of the participants.

**Figure 1:**
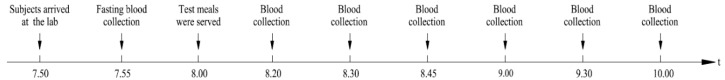
Glycemic response test flow.

On each trial day, the subjects arrived at the laboratory at 7.50 a.m. and took a rest for 5 min before the baseline blood glucose test started at 7.55 a.m. The test meals were provided by a person who was not involved in data analysis to the subjects at 8:15 a.m. they were instructed to consume a test food and a small glass of water (to balance the size of the meals) and finish the meal within 20 min. The finger prick blood samples were taken at 20, 30, 45, 60, 90 and 120 min following the start of the meal (Figure 1). To avoid possible plasma dilution, the second drop of blood was used for testing. Subjects could read a book or use a laptop as long as they remain seated during test sessions. They were not permitted to eat food that was not related to the experiment or to discuss anything about food. Plasma blood glucose was determined with an ONETOUCH^®^ Ultra^®^ System (LifeScan Inc., Milpitas, CA, USA). According to the handbook, the accuracy of the system was assessed by comparing blood glucose results obtained by patients with diabetes with those obtained using an YSI Model 2300 Glucose Analyzer. The coefficient of variation (CV, CV = 100 × SD/mean) of within run blood glucose was lower than 3.2%, and the CV of total blood glucose was lower than 4.4%.

### 2.4. In Vitro Determination of Carbohydrate Digestibility

In vitro digestion of carbohydrate was measured by a modified Englyst method [24]. The diluted amyloglucosidase solution was prepared by adding 2.8 mL of amyloglucosidase (3000 U/mL, EC 3.2.1.3.; Megazyme International Ireland Ltd., Bray, Ireland) to 7.2 mL of water. The α-amylase solution was prepared by adding 3.0 g of porcine pancreatic α-amylase (150 U/mg; P1625, Sigma-Aldrich, St. Louis, MO, USA) into each four centrifuge tubes with 17 mL of water at 37 °C. The mixture was stirred magnetically for 10 min and centrifuged for 10 min, and then 13.5 mL of supernatant was taken from each tube and mixed with 6 mL of diluted amyloglucosidase. Sodium acetate buffers (0.1 mol/L, pH 5.2) were prepared by dissolving 13.6 g of sodium acetate trihydrate in 250 mL of saturated benzoic acid solution and adding approximately 750 mL of water. To simulate the chewing process, a portion of cooked test food was put in a Midea High-Performance Blender (Midea Group, Guangdong, China) and mashed at the lowest speed (3000 r/min) for 15 s. Five grams of mash was mixed with 50 mg of guar gum powder, two big and two small glass balls, and pH was modified to 5.2. After 5 mL of mixed enzyme solution was added, the mixture was quickly incubated in a shaking water-bath machine (37 °C, 200 strokes/min). To stop enzyme activity, 95% of ethanol was added at 0, 2, 5, 10, 20, 60, and 120 min, respectively. Then released glucose was assayed with GAGO20 Sigma Glucose assay kits.

### 2.5. Statistical Analysis

Total areas under the curve of postprandial GR (iAUC) and incremental peak blood glucose values were calculated. The iAUC values of postprandial GR were determined using the trapezoidal method and areas beneath the fasting blood glucose level were ignored. GI values were defined as the iAUC_0–120_ value generated by 50 g of available carbohydrate of the test food expressed as a percentage of the response generated by 50 g of glucose as reference food. Regarding in vitro carbohydrate digestion, rapidly digestible starch (RDS), SDS, resistant starch (RS) and hydrolysis indexes (HI) [24] were calculated. HI values were defined as the total areas under the curve of hydrolysis generated by test food expressed as a percentage of the hydrolysis generated by the same mass of WR as reference food.

Data analysis was performed with SPSS 21.0 software (SPSS Inc., Chicago, IL, USA). The Kolmogorov-Smirnov test was involved to test whether data were normally distributed. A natural logarithmic transformation was expected to normalize the non-normal distributed data. For blood glucose and starch digestibility data, repeated-measure analysis of variance (RMANOVA) and Tukey’s test were used to examine the difference, *p* < 0.05. Data were presented as means (standard errors, SE) or means (standard deviations, SD) where appropriate. Figures were generated with Origin 9.1 (OriginLab Inc., Northampton, MA, USA).

## 3. Results

### 3.1. Subject Enrolment

**Figure 2:**
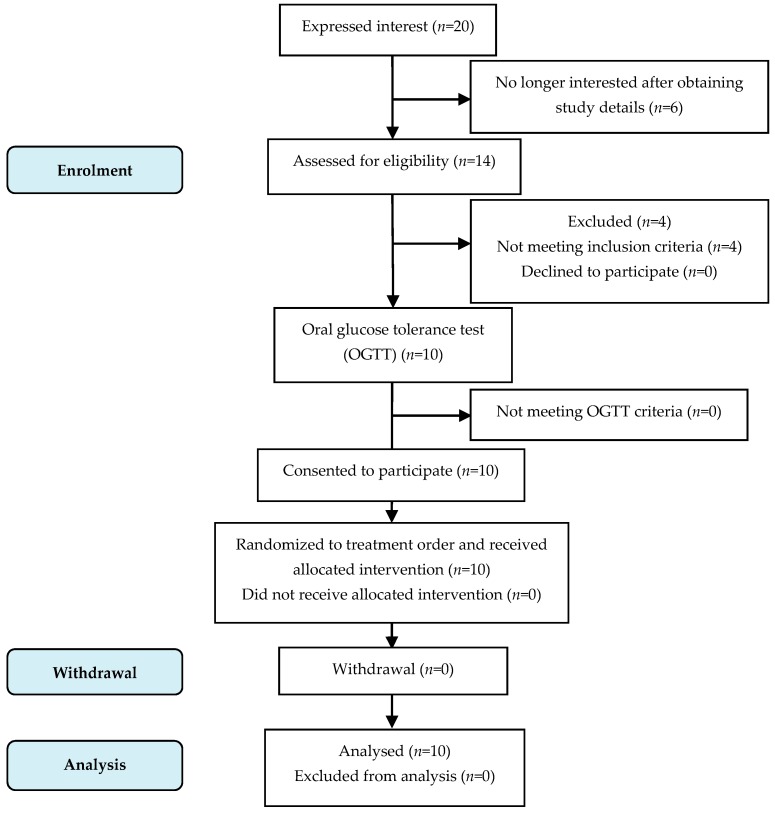
Consolidated standards of reporting trial (CONSORT) flow diagram of the study subjects.

The study subject flow of blood glucose tests is shown in Figure 2. Ten subjects completed all the test sessions, and all their data were included.

### 3.2. Subject Characteristics

Subject baseline characteristics are shown in Table 2. The mean value of fasting blood glucose concentration was 4.8 (SE 0.2). In terms of fasting blood glucose values, there was no difference among all test foods and reference foods.

### 3.3. Blood Glucose

**Figure 3:**
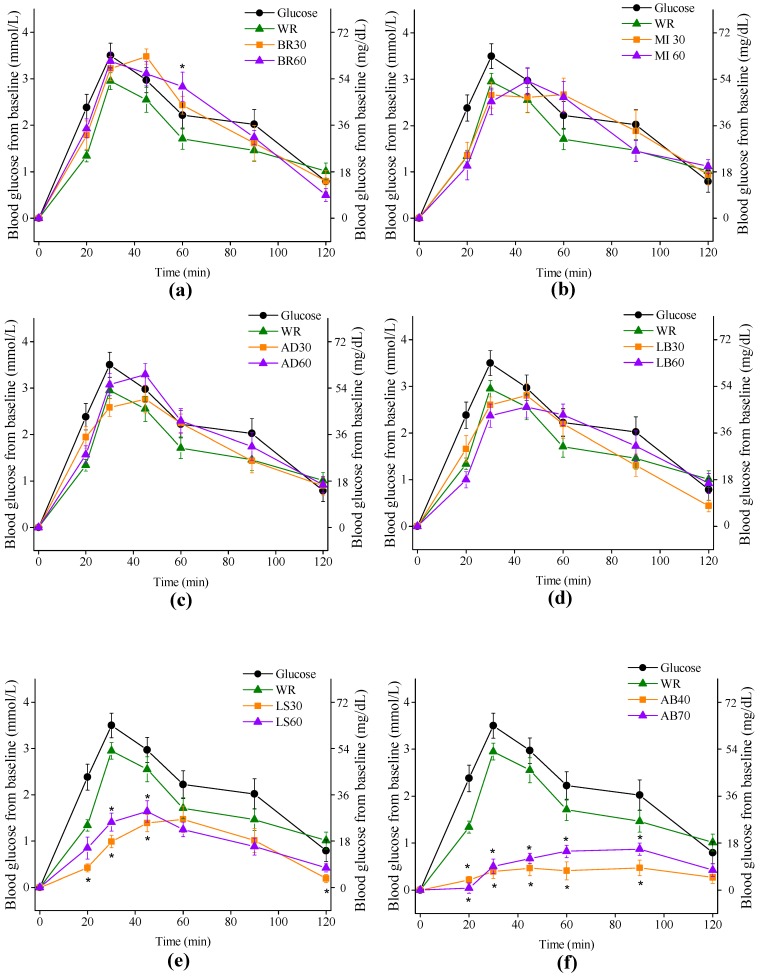
Blood glucose changes from baseline for test foods. WR, white rice cooked for 30 min; (**a**) waxy black rice cooked for 30 min (BR30), waxy black rice cooked for 60 min (BR60); (**b**) millet cooked for 30 min (MI30), millet cooked for 60 min (MI60); (**c**) adlay cooked for 30 min (AD30), adlay cooked for 60 min (AD60); (**d**) dried lily bulb cooked for 30 min (LB30), dried lily bulbs cooked for 60 min (LB60); (**e**) lotus seed cooked for 30 min (LS30), lotus seeds cooked for 60 min (LS60) (**f**) adzuki bean cooked for 40 min (AB40), adzuki bean cooked for 70 min (AB70). Values are the mean changes in blood glucose levels from baseline, *n* = 10, with their standard errors represented by vertical bars. Differences between test foods and white rice are shown (* *p* < 0.05).

The glucose responses for all test foods are shown in Figure 3. The adlay cooked for 30 and 60 min (abbreviated as AD30 and AD60), the dried lily bulb cooked for 30 and 60 min (abbreviated as LB30 and LB60), and the waxy black rice cooked for 30 min (BR30) achieved peak values at 45 min, while the waxy black rice cooked for 60 min (BR60), the WR and glucose control achieved showed incremental peak values at 30 min. The lotus seed cooked for 60 (LS60) and 30 min (LS30) had their incremental peak values at 45 and 60 min, respectively. The millet meals (abbreviated as MI30 and MI60) showed plateau-style glucose levels through 30 min to 60 min after meal. The adzuki bean meals cooked for 40 and 70 min (abbreviated as AB40 and AB70), however, did not show typical peak of glucose level.

Both AB meals elicited remarkably lower glucose response than any other test foods did. Compared to the WR, AB40, and AB70 had lower blood glucose level on all time points except 120 min. The lotus seed meals also manifested low glycemic response as the LS30 had significantly lower incremental values of blood glucose at 20, 30 and 45 min (0.2 (SE 0.1) mmol/L, *p* = 0.013; 1.4 (SE 0.1) mmol/L, *p* = 0.000; 1.5 (SE 0.1) mmol/L, *p* = 0.009, respectively), while the LS60 had significantly lower incremental values of blood glucose at 30 min and 45 min (1.4 (SE 0.2) mmol/L, *p* = 0.000; 1.6 (SE 0.2) mmol/L, *p* = 0.044, respectively), compared to that of the WR. The waxy black rice cooked for 60 min (BR60) had a higher incremental value of blood glucose at 60 min (2.8 (SE 0.3) mmol/L, *p* = 0.022), compared with WR.

The cooked LS and AB could be classified as low-GI foods (GI < 55) and had significant differences with any other test foods in terms of GI values, iAUC_0–60_, iAUC_0–120_ and peak values of glucose, while the GI values of cooked waxy black rice, adlay, millet, dried LBs were high-GI foods (GI < 70) and were comparable with WR in terms of GI values, iAUC_0–60_, iAUC_0–120_ and peak values, regardless of cooking treatment (Table 3). A negative correlation was observed between GI values and total (*r* = 0.680, *p* = 0.011) and insoluble dietary fiber (*r* = 0.690, *p* = 0.009). Greater cooking duration resulted in higher GI values for all test foods expect for millet, but not significantly. The mean reference CV (glucose as reference) of iAUC_0–120_ of the subject group was 28.9, which was inside the upper recommended threshold of 30 [25].

### 3.4. In Vitro Starch Digestibility

**Figure 4:**
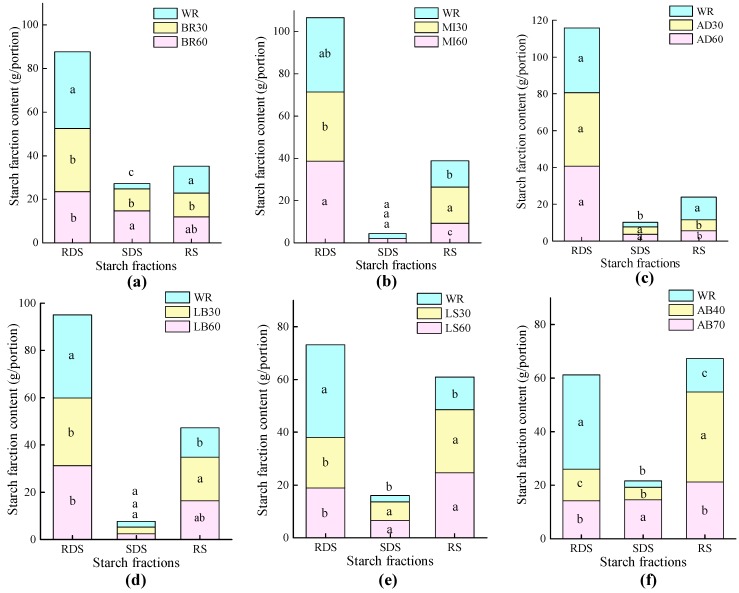
The constituents of starch fractions of test foods (Mean values with their standard deviations, *n* = 6). RDS, rapidly digestible starch; SDS, slowly digestible starch; RS, resistant starch; WR, white rice cooked for 30 min; (**a**) BR30 and BR60, waxy black rice cooked for 30 and 60 min; (**b**) MI30 and MI60, millet cooked for 30 and 60 min; (**c**) AD30 and AD60, adlay cooked for 30 and 60 min; (**d**) LB30 and LB60, dried lily bulb cooked for 30 and 60 min; (**e**) LS30 and LS60, lotus seed cooked for 30 and 60 min; (**f**) AB40 and AB70 adzuki bean cooked for 40 and 70 min. Differences of RDS, SDS, and RS between test foods and white rice are shown with unlike superscript letters, a,b,c (*p* < 0.05).

In case of the BR, LS, LB and AB meals, RDS fractions were significantly lower than that of WR (*p* < 0.05) (Figure 4). Except for the MI60, BR30, AD30, and AD60, the RS fractions of the test foods were significantly higher than that of WR (*p* < 0.05).

The HI of AB40 was significantly lower than those of other test foods. The HIs of LS30, LS60, and AB70 were significantly lower than others except for AB40 (Table 4). AD30 and AD60 had highest HIs, followed by MI30, MI60, BR30, and BR60. There was a negative correlation between HI values and total (*r* = 0.695, *p* = 0.008) and insoluble dietary fiber (*r* = 0.624, *p* = 0.023). In addition, fairly good consistency between HI and GI (*r* = 0.864, *p* = 0.000), RDS fractions and GI (*r* = 0.777, *p* = 0.001), RS fractions and GI (*r* = 0.853, *p* = 0.000) was observed.

## 4. Discussions

The results of the present study do not completely agree with our hypothesis that all the test non-cereal foods would have a positive effect on postprandial GR. Among the 3 starchy foods tested in the study, only the lotus seed (LS) meals could be regarded as low-GI food, compared to the adzuki bean (AB) meals. However, other test foods, including the cooked dried lily bulb (LB) and adlay (AD), waxy black rice (BR), and millet (MI), were as high-GI foods as the white rice (WR), regardless of the cooking duration.

The acute GR of AD [19] and AB, hot-water extract included [26], were sparsely reported previously, but to our knowledge, the current study was the first study to investigate the domestically prepared LS, LB, and BR on GR of human subjects using the standard test protocol.

It has been reported that basmati rice [27] and brown rice [19] could elicit high GR after pre-soaking, while AD gave a medium GI of 55 when cooked without pre-soaking [19]. The foxtail millet was reported to have a high GI of 93.6 as millet porridge and a moderate GI of 64.4 as millet meal cooked without pre-soaking [28]. In animal studies, feeding millet improved insulin sensitivity and blood lipid metabolism in type 2 diabetic mice [29], it was expected that millet might be beneficial to the prevention of type 2 diabetes. However, in default domestic practice of East Asia families, the whole grains are pre-soaked overnight as we did in this study to make the cooking process easier and improve the taste and mouthfeel [19], which may increase RDS fraction and elevate the GR.

Despite being a whole grain food, the cooked BR showed startling high GIs of 100 and 109, which might be explained by its being a waxy cereal. The starch of waxy varieties of cereals consists of almost pure amylopectin [30], while high percentage of amylopectin is usually associated with low RS, rapid digestion process [30] and high GR [31]. It was reported that compared with non-waxy rice flour, waxy rice flour had lower pasting temperature, higher peak viscosity and reduced resistance to breakdown [32]. The waxy millet and waxy proto-millet were also reported to have extreme high GIs of 108 and 111, much higher than their non-waxy counterparts [21].

It is worth noticing that the LS meals had low GI value even after pre-soaking treatment. Like AD, LS had long been an ingredient in Asian diet. The LS and AD had comparable content of carbohydrate, protein, and fiber, but the LS meals elicited much lower GR than the AD did. The LS starch has a high amylose content of around 40% and is prone to retrogradation [33], which made it a very high RS carbohydrate food with good prebiotic properties [34]. Wu et al. [35] previously reported a GI of LS as 62, though in that GI test only the blood glucose values of 30, 60, 120, and 180 min after meal were included, which was not in accord with the international standard GI test protocol (ISO/FDIS 26642:2010 Food products—Determination of the GI and recommendation for food classification). An animal study indicated that some constituents in LS might have hypoglycemic effect in diabetes rats [36].

The AB had the lowest GR among the test foods in the present study. Even after the overnight pre-soaking and 70-min cooking, the AB70 test sample elicited a GI value as low as 29. The AB starch was characterized by a large starch particle size, highly homogeneous and dense starch granule, relatively few branches in amylose and amylopectin, high transition temperature during gelatinization, low susceptibilities to enzymes [37], low swelling power and easy retrogradation [38]. The contact structure and rigid cell wall of AB could effectively inhibit the water absorption and the hydration of the starch, which makes its natural structure difficult to be disrupted by prolonged heating. Moreover, the AB is rich in phytic acid and polyphenolic compounds including tannins, phenolic acids and flavonoids [39] that may confer anti-diabetic effects [40]. There were reports that a type of convenient food made of extruded AB improved glycemic regulation in patients with type 2 diabetes [41] and that hot-water extract of AB attenuated hyperglycemia and hyperinsulinemia in spontaneously diabetic mice [26].

In the present study, cooking time made no difference with respect to the GI value of the test samples, as the combination of overnight pre-soaking and a relatively short cooking time as 30 min could ensure high RDS fractions in millet, BR, AD, and LB samples. It seems that the pre-soaking treatment fully overcame the physical barrier of botanical integrity of whole grains, which otherwise should have rendered a physical barrier of water absorption and helped to reduce the level of gelatinization [42] and mitigate GR [19]. High levels of RDS could accelerate the enzymatic hydrolysis process [43] and the absorption of glucose, which eventually resulted in high GR [44]. However, the AB displayed a very slow in vitro carbohydrate digestion, low RDS, and high RS fractions, which could contribute to lower postprandial GRs and insulin sensitivity improvements [45]. It was reported that starchy foods with low RDS fractions and high RS fractions contributed to lower postprandial GRs and insulin sensitivity improvement [46]. A significant reduction of GR and insulin response was observed after the consumption of foods containing 8.0–18.9 g of RS [47,48]. In the present study, the RS intake of one portion of AB40, AB70, LS30, and LS60 was 33.6 g, 21.2 g, 23.8 g and 24.6 g respectively, which could ensure a mitigating effect on GR.

Accumulating evidence indicated that substituting whole grains and pulses for refined grains in rice consumption culture renders a feasible approach to managing the risk of chronic non-communicable diseases such as diabetes and cardiovascular disease, especially for those high-risk groups who have central obesity and insulin resistance. The favorable effects of adding whole grain and pulses into rice diet include lowering triglycerides and systolic pressure [49], reducing the risk of abdominal obesity and abnormal fasting glucose [50], decreasing low density lipoprotein cholesterol and fasting glucose [51], and mitigating the prandial glycemic response and insulinemic response [52,53].

However, as markers of carbohydrate quality, the GI and glycemic load (GL) failed to prove consistent high-quality evidence for its eligible application to chronic disease prevention or dietary management. According to the latest systematic reviews and meta-analysis, the GI could explain only 5% of the health benefit of dietary fiber intake from whole grains [54]. The inconsistency results in relevant cohorts and trials might partly be attributed to the discrepancy between the GI values in GI database or literatures and the real GIs of food prepared in real life, as shown in the current study.

Nevertheless, even with high GI values after pre-soaking and cooking, the non-cereal starchy seeds as well as whole grains are better source of carbohydrates, if taking their nutrient density into account. Given the calorie and carbohydrate intake unchanged, the addition of these ingredients into rice meal is still recommendable for non-diabetic people as they generally have higher potassium, B vitamins and dietary fiber content [7] compared with polished rice, as the latest evidence proved that the fiber intake from whole grains might be the decisive factor in reducing all-cause mortality [54].

The present study has some limitation. Firstly, the study was only conducted in healthy young women with normal BMI, and however people with impair glucose tolerance or metabolic diseases were excluded. It was reported that there was no significant difference between male and female participants in terms of glycemic response [55] and most of the responder were female, so only female participants were included in the present study. In view of the effects of cooked whole grains, legumes and other starchy foods on postprandial GR might be more significant in people with central obesity or pre-diabetes, so further study in glycemically compromised subjects is expected. Secondly, we did not make the comparison between samples of non-soaked and pre-soaked in terms of GR, so the optimal cooking procedures of the LB are still to be tested. Thirdly, as this study was just a pilot study, insulinemic responses and gastrointestinal hormones, which would have provided more explanations of mechanisms, were not investigated. Finally, only the acute effect and GI were evaluated in this study, while the long-term effect of the three non-cereal starchy foods as ingredient of staple food on glycemic management needs to be explored.

In conclusion, the results of the current study suggested that: (1) despite being whole grains, the waxy type of cereals and the pre-soaked cereals can be very high-GI food; (2) pulses such as the AB can maintain low GI even after a long time pre-soaking and prolonged cooking; (3) the LS is a valuable low-GI food ingredient in staple food, which behaved more like pulses in terms of GR, while the AD and LB were more like millet, and strongly depend on cooking practice with respect to GI values. Careful choice of whole grain materials, minimized pre-soaking, and moderate cooking may be critical factors for successful glycemic management for people of impaired glucose management. Since limited data were available regarding the preparation practice on the metabolic markers of whole grains and starchy foods, further studies of both acute and long-term effects on this aspect are needed.

## Figures and Tables

**Table 1:** Composition of test foods ^1^.

g (Fresh Weight/Portion)	ACHO ^2^	Protein	Fat	Total Dietary Fiber	Insoluble Dietary Fiber	Soluble Dietary Fiber	Meal Size ^3^	Energy (kcal)
Glucose	50.0	-	-	-	-	-	250	199.0
White rice	50.0	4.5	0.5	0.7	0.7	0	230	221.5
Waxy black rice	50.0	4.7	1.8	2.6	2.4	0.2	230	233.9
Millet	50.0	6.5	2.2	1.2	1.1	0.1	230	244.6
Adlay	50.0	12.2	2.5	1.5	1.4	0.1	230	270.0
Dried lily bulb	50.0	6.8	0.7	2.0	1.9	0.1	230	232.4
Lotus seed	50.0	12.0	0.6	1.5	1.5	0	230	252.2
Adzuki bean	50.0	18.4	0.5	6.4	5.7	0.7	230	276.8

^1^ Nutritional data were obtained from manufactures. ^2^ ACHO, available carbohydrate. ^3^ Including the water used for cooking or balancing the weight.

**Table 2:** Subject baseline characteristics (Mean values with their standard deviations (SD), *n* = 10).

Characteristics	Value	
	Mean	SD
Number of participants (*n*)	10	-
Age (year)	20.7	2.3
Body height (cm)	163.4	6.4
Body weight (kg)	58.9	6.2
BMI (kg/m^2^)	22.0	2.1
Fat mass (%)	23.6	4.5
Basal metabolism rate (BMR) (kcal/day)	1316	123.8
Fasting blood glucose (mmol/L)	4.8	0.2

**Table 3:** Postprandial glycemic characteristics of test foods in 120 min (Mean values with their standard errors (SE), *n* = 10).

Sample	Incremental Peak of Glucose (mmol/L)		iAUC_0–60_ (mmol/L·2 h)		iAUC_0–120_ (mmol/L·2 h)		GI		Classification
	Mean	SE	Mean	SE	Mean	SE	Mean	SE	-
Glucose	3.6 ^a^	0.3	140 ^a^	11.3	246.4 ^a^	22.6	100	-	High
WR	3.2 ^a^	0.2	108.1 ^b^	6.9	192.7 ^a^	14.3	83 ^b^	9	High
BR30	3.7 ^a^	0.2	137.5 ^a^	8.4	234.6 ^a^	22.3	100 ^ab^	10	High
BR60	3.7 ^a^	0.2	141.6 ^a^	7.0	246.8 ^a^	13.5	109 ^a^	12	High
MI30	3.5 ^a^	0.2	112.8 ^b^	7.2	223.4 ^a^	21.6	93 ^ab^	8	High
MI60	3.4 ^a^	0.2	112.4 ^b^	8.9	211.9 ^a^	16.3	89 ^ab^	6	High
AD30	3.1 ^a^	0.2	119.6 ^b^	6.3	209.4 ^a^	14.0	91 ^ab^	10	High
AD60	3.7 ^a^	0.2	128.5 ^ab^	6.5	228.8 ^a^	14.8	100 ^ab^	11	High
LB30	3.1 ^a^	0.2	115.8 ^b^	7.9	194.6 ^a^	17.6	83 ^b^	9	High
LB60	3.1 ^a^	0.2	101.0 ^b^	8.2	202.2 ^a^	19.3	85 ^b^	7	High
LS30	1.8 ^b^	0.2	50.6 ^c^	4.0	105.8 ^b^	9.3	45 ^c^	5	Low
LS60	1.9 ^b^	0.2	61.4 ^c^	8.8	115.8 ^b^	11.5	51 ^c^	7	Low
AB40	0.7 ^c^	0.1	19.5 ^d^	2.9	45.8 ^c^	6.5	21 ^d^	4	Low
AB70	1.1 ^c^	0.1	21.8 ^d^	4.2	65.0 ^c^	5.6	29 ^cd^	4	Low

The iAUC_0–60_ or iAUC_0–120_ for postprandial GR was determined for the time interval in one hour or two hours using the trapezoidal method, ignoring the area beneath the fasting level; GI, glucose index; WR, white rice cooked for 30 min; BR30 and BR60, waxy black rice cooked for 30 and 60 min; MI30 and MI60, millet cooked for 30 and 60 min; AD30 and AD60, adlay cooked for 30 and 60 min; LB30 and LB60, dried lily bulb cooked for 30 and 60 min; LS30 and LS60, lotus seed cooked for 30 and 60 min; AB40 and AB70 adzuki bean cooked for 40 and 70 min. Values are the mean glycemic characteristics of test foods, *n* = 10, with their standard errors. ^a,b,c,d^ Mean values within a column with unlike superscript letters are significantly different (*p* < 0.05).

**Table 4:** The hydrolysis index of test foods (Mean values with their standard deviations (SD), *n* = 6).

Sample	HI (%)		Sample	HI (%)	
	Mean	SD		Mean	SD
WR	100	-	LB30	81.9 ^d^	1.9
BR30	90.3 ^c^	3.2	LB60	87.7 ^cd^	0.8
BR60	89.3 ^c^	0.6	LS30	61.1 ^e^	2.3
MI30	93.1 ^c^	1.6	LS60	58.6 ^e^	1.0
MI60	107.3 ^b^	2.1	AB40	37.7 ^f^	1.0
AD30	113.9 ^a^	2.0	AB70	55.9 ^e^	3.0
AD60	116.2 ^a^	2.3			

HI, hydrolysis index; WR, white rice cooked for 30 min; BR30 and BR60, waxy black rice cooked for 30 and 60 min; MI30 and MI60, millet cooked for 30 and 60 min; AD30 and AD60, adlay cooked for 30 and 60 min; LB30 and LB60, dried lily bulb cooked for 30 and 60 min; LS30 and LS60, lotus seed cooked for 30 and 60 min; AB40 and AB70, adzuki bean cooked for 40 and 70 min. ^a,b,c,e,f^ Mean values within a column with unlike superscript letters are significantly different (*p* < 0.05).

## Supplementary Materials

The following are available online at https://www.mdpi.com/2072-6643/11/3/634/s1, Satiety of six domestically cooked starchy foods.
