# Supplementary material for: Acute Effects of Three Cooked Non-Cereal Starchy Foods on Postprandial Glycemic Responses and in Vitro Carbohydrate Digestion in Comparison with Whole Grains: A Randomized Trial
Source: Nutrients. 2019 Mar 15;11(3):634. doi: 10.3390/nu11030634 (PMC6471337; doi:10.3390/nu11030634)
Supplement: Supplementary file 1 [file nutrients-11-00634-s001.pdf]

Supplementary Materials:

Satiety of six domestically cooked starchy foods

Methods

Visual analogue scale (VAS) [1] was used to measure fullness ratings in the satiety test. The subjects rated on a splash on each scale how full they felt from -100 to 100 points, where -100 points represented ''Greatest imaginable hunger'' and 100 points represented ''Greatest imaginable fullness''. The satiety test was taken before the meal and at 20, 30, 45, 60, 90, 120, 150, 180, 210 and 240 min after the meal.

Statistical Analysis

Data analysis was performed with SPSS 21.0 software (SPSS Inc. Chicago, IL, USA). The Kolmogorov-Smirnov test was involved to test whether data were normally distributed. A natural logarithmic transformation was expected to normalise the non-normal distributed data. For satiety data, repeated-measure analysis of variance (RMANOVA) and Tukey's test were used to examine the difference, $p < 0.05$. Data were presented as means (standard errors, SE).

Results

The fullness rating for all test foods is shown in Figure. Although starchy foods failed to show higher peak fullness rates compared to the glucose control, all of them except for BR30 and BR60 had significantly lower fullness rates at 240 min (the end of the satiety test). Similarly, the peak fullness rates of starchy foods were not significantly higher than that of white rice, but MI30, AD30, LB30, LB60, LS30, AB40 and AB70 showed significantly lower fullness rates at 240 min compared with the rice reference. However, there was no difference between test foods cooked under different duration.

Discussions

We found that although some starchy foods, especially BR30, BR60 and AD30, had higher glucose responses and GI values compared with white rice and glucose control, they showed higher satiety, which means they could help to delay the subsequent meal or reduce energy intake at the subsequent meal. On the one hand, the high satiety of starchy foods may be attributed to dietary fibre [2]. Dietary fibre could improve satiety via increased mastication or changes in satiety-related gut hormones [3]. Most pulses, such as adzuki beans used in the present study, are rich in soluble fibre [4]. Soluble fibre is reported to have the potential to form a viscous gel matrix in the gut and slow gastric emptying, which may cause a greater feeling of fullness [5]. Additionally, insoluble fibre, which is widely found in the starchy foods used in the current study, is also found to make contribution to satiety [6,7]. On the other hand, higher resistant starch content may also play an important role in improving satiety by regulating gut hormones [8] and delaying gastric emptying [9].

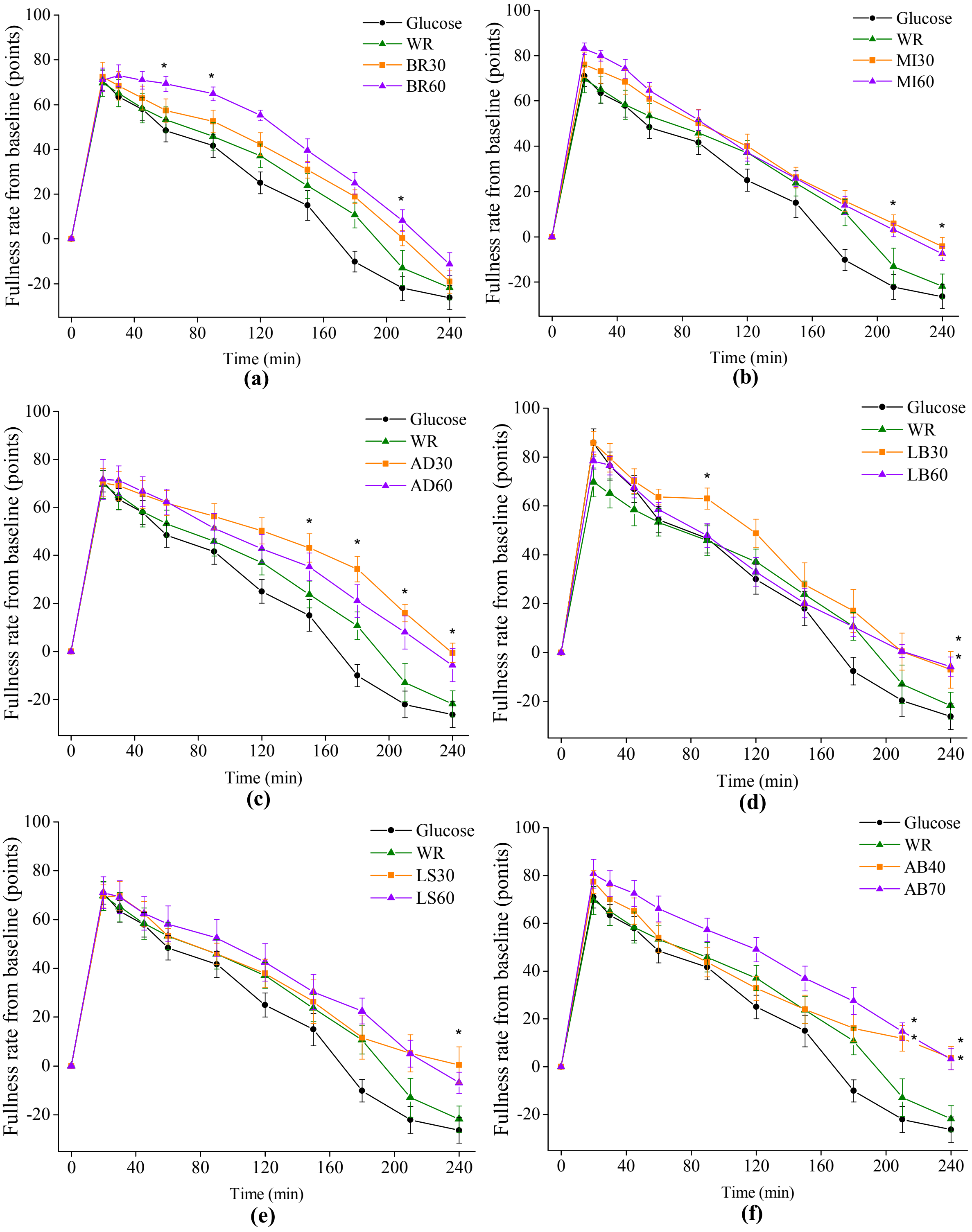

Figure. Fullness rate changes from baseline for test foods. WR, white rice cooked for 30 min; (a) waxy black rice cooked for 30 min (BR30), waxy black rice cooked for 60 min (BR60); (b) millet cooked for 30 min (MI30), millet cooked for 60 min (MI60); (c) adlay cooked for 30 min (AD30), adlay cooked for 60 min (AD60); (d) dried lily bulb cooked for 30 min (LB30), dried lily bulbs cooked for 60 min (LB60); (e) lotus seed cooked for 30 min (LS30), lotus seeds cooked for 60 min (LS60) and (f) adzuki bean cooked for 40 min (AB40), adzuki bean cooked for 70 min (AB70). Values are the mean changes in fullness rate from baseline, n = 10, with their standard errors represented by vertical bars. Differences between test foods and white rice are shown (*$p < 0.05$).

References

1. Merrill, E.P.; Cardello, A.V.; Kramer, F.M.; Schutzc, H.G.; Lesherb, L.L. The development of a perceived satiety index for military rations. *Food. Qual. Prefer.* **2004**, 15, 859-870.
2. Clark, M.J.; Slavin, J.L.. The effect of fiber on satiety and food intake: a systematic review. *J. Am. Coll. Nutr.* **2013**, 32, 200-211.
3. Burton-Freeman B.; Davis, P.A.; Schneeman, B.O. Plasma cholecystokinin is associated with subjective measures of satiety in women. *Am. J. Clin. Nutr.* **2002**, 76, 659-667.
4. Messina, V. Nutritional and health benefits of dried beans. *Am. J. Clin. Nutr.* **2014**, 100, 437S-442S.
5. Hoad, C.L.; Rayment, P.; Spiller, R.C.; Marciani, L.; Alonso, B.C.; Traynor, C.; Mela, D.J.; Peters, H.P.; Gowland, P.A. In vivo imaging of intragastric gelation and its effect on satiety in humans. *J. Nutr.* **2004**, 134, 2293-2300.
6. Samra, R.A.; Anderson, G.H. Insoluble cereal fiber reduces appetite and short-term food intake and glycemic response to food consumed 75 min later by healthy men. *Am. J. Clin. Nutr.* **2007**, 86, 972-979.
7. Levine, A.S.; Tallman, J.R.; Grace, M.K.; Parker, S.A.; Billington, C.J.; Levitt, M.D. Effect of breakfast cereals on short-term food intake. *Am. J. Clin. Nutr.* **1989**, 50, 1303-1307.
8. Keenan, M.J.; Zhou. J.; McCutcheon, K.L.; Raggio, A.M.; Bateman, H.G.; Todd, E.; Jones, C.K.; Tulley, R.T.; Melton, S.; Martin, R.J.; Hegsted, M. Effectsof resistant starch, a non-digestible fermentable fiber, on reducing body fat. Obesity **2006**, 14, 1523-1534.
9. Nilsson, A.C.; Ostman, E.M.; Holst, J.J.; Bjorck, I.M. Including indigestible carbohydrates in the evening meal of healthy subjects improves glucose tolerance, lowers inflammatory markers, and increases satiety after a subsequent standardized breakfast. *J. Nutr.* **2008**, 138, 732-739.
